# Diagnostic yield of *Legionella pneumophila* PCR across specimen types during a community outbreak

**DOI:** 10.1128/jcm.01116-26

**Published:** 2026-10-12

**Authors:** Padman Jayaratne, Carson K. L. Lo, Candy Rutherford, Adam S. Komorowski, Daniela Leto, Marek Smieja, Mohammad Rubayet Hasan

**Affiliations:** 1 Hamilton Regional Laboratory Medicine Program 709550, Hamilton, Canada; 2 Department of Pathology and Molecular Medicine, McMaster University 3710, Hamilton, Canada; 3 Research Institute of St. Joe's Hamilton 539740 , Hamilton, Canada; 4 Division of Infectious Diseases, Department of Medicine, McMaster University 3710, Hamilton, Canada; Vanderbilt University Medical Center, Nashville, Tennessee, USA

**Keywords:** *Legionella pneumophila*, Legionnaires' disease, respiratory tract infections, PCR

## LETTER

*Legionella pneumophila* is an important cause of community-acquired and healthcare-associated pneumonia. Diagnostic recommendations combine lower respiratory tract culture or molecular methods and urinary antigen testing (UAT) (1, 2). Although UAT is rapid and non-invasive, it primarily detects *L. pneumophila* serogroup 1 and may fail to identify infections caused by other serogroups (3–5). Molecular testing of respiratory specimens can improve case detection, but its diagnostic yield varies according to specimen type (6). Bronchoalveolar lavage (BAL) is generally considered the preferred respiratory specimen for *Legionella* PCR; however, bronchoscopy is an invasive procedure that may not be feasible or clinically indicated in many patients with suspected *Legionella* pneumonia (5, 7). Less invasive specimens, such as sputum, nasopharyngeal swabs (NPS), and urine, are easier to obtain, but data directly comparing PCR detection across different clinical specimen types remain limited, particularly during outbreak investigations (6, 8, 9).

This study was conducted in the context of a *Legionella* outbreak in Hamilton, Ontario, Canada, between 4 May and 26 May 2026. All laboratory-confirmed cases required hospitalization, and patients ranged in age from their late 30s to early 90s. Despite extensive investigation, including assessment of potential environmental sources such as cooling towers, no common source of the outbreak was identified (10). In this study, we evaluated *L. pneumophila* PCR detection in BAL, sputum, NPS, and urine specimens from UAT-positive patients. Cases were identified by positive UAT results obtained at Public Health Ontario’s reference laboratory. BAL and sputum submitted for bacterial culture, as well as NPSs submitted for respiratory virus testing at the Hamilton Regional Laboratory Medicine Program (HRLMP), were retrospectively retrieved. Not all specimen types were available from every patient, reflecting routine clinical practice. A laboratory-developed multiplex real-time *Legionella* PCR assay was performed targeting (i) *Legionella* spp. *ssrA* gene, (ii) *L. pneumophila mip* gene, and (iii) lambda phage DNA as an internal control. The assay was previously analytically validated for BAL specimens; testing of sputum, NPS, and urine was investigational and not reported for clinical management. Detailed methods are provided in the supplemental material.

**Fig 1:**
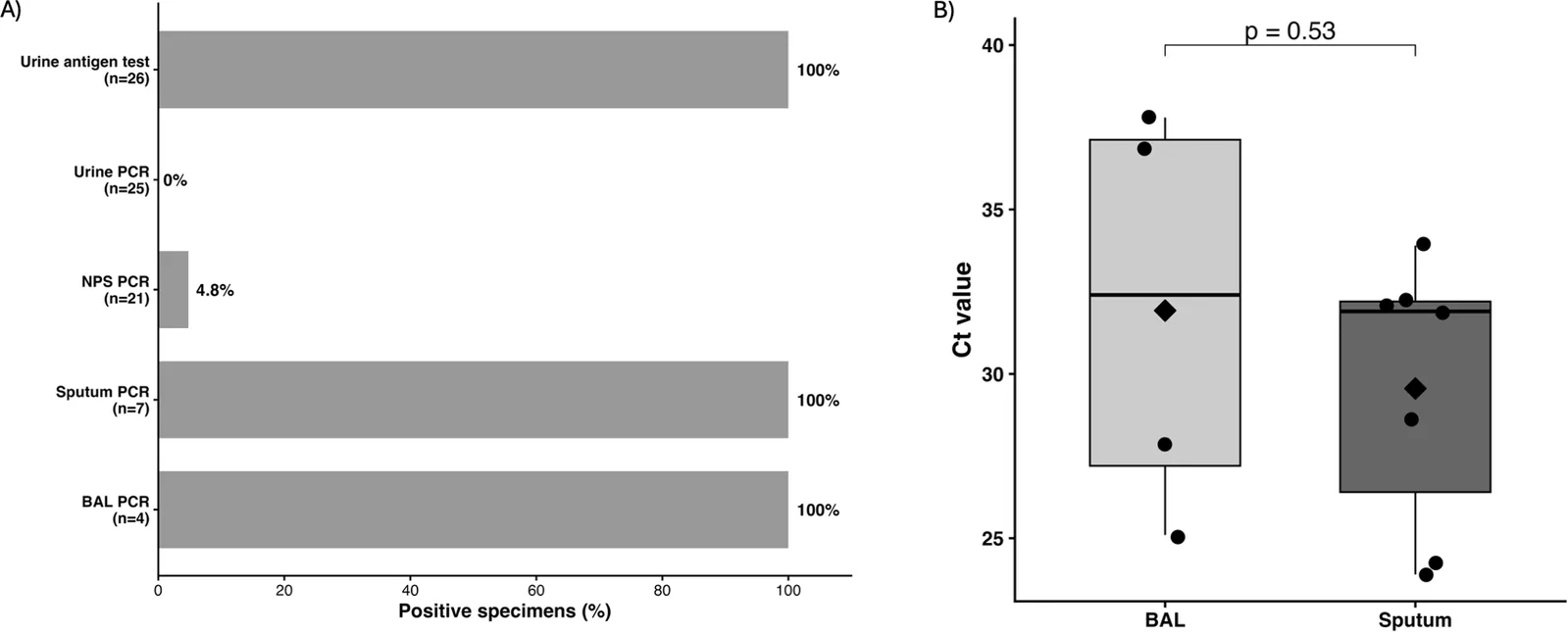
Comparison of different specimen types for *Legionella pneumophila* PCR during a community outbreak. (**A**) Diagnostic yield of *L. pneumophila* PCR using different specimen types compared with the urine antigen test. (**B**) Comparison of *L. pneumophila* PCR Ct values between sputum and bronchoalveolar lavage (BAL) specimens. *P* value calculated using a two-tailed Mann–Whitney U test.

Twenty-six UAT-positive patients were included. Urine PCR was negative in all specimens in which it was performed (*n* = 0/25, 0%), whereas NPS PCR was positive in 1 of 20 (5%) evaluated patients (Fig. 1A). Sputum and BAL PCRs were positive in all patients in which they were performed (7/7 [100%] and 4/4 [100%], respectively). Median Ct values did not differ significantly between sputum and BAL (31.9 [range, 23.9–33.9] vs 32.4 [range, 25.1–37.8]; two-tailed Mann–Whitney U test, *P* = 0.53) (Fig. 1B). Paired BAL and sputum specimens were available for only two patients, both of whom had concordant positive results from sputum and BAL specimens. NPS PCR was negative in eight patients with a positive BAL or sputum specimen. The only NPS-positive patient had a negative urine PCR, with no lower respiratory tract specimen submitted.

To further assess assay performance in sputum, 10 additional sputum specimens from patients with negative UAT results were tested, and all were PCR negative. Taken together, sputum PCR demonstrated 100% concordance with UAT results among the 17 specimens tested by both methods.

*L. pneumophila* PCR detection differed markedly by specimen type. All tested sputum and BAL specimens yielded positive results in patients with confirmed Legionnaires’ disease. While BAL remains the preferred specimen type (7), our limited data suggest that sputum may be a practical alternative when bronchoscopy is not indicated. Conversely, NPS detection was very limited, consistent with the lower respiratory tract localization of *L. pneumophila* infection, and urine PCR had no apparent utility despite universally positive UAT results. The absence of detectable *L. pneumophila* DNA in urine suggests that urinary antigen detection and molecular detection target fundamentally different analytes and should not be considered interchangeable.

Our study has several limitations. First, the sample size was small, limiting the precision and generalizability of our findings and precluding definitive conclusions regarding the performance of sputum, NPS, and urine specimens. Second, specimens were incompletely paired across specimen types, and sputum, NPS, and urine testing was not formally validated against a reference standard. In addition, molecular testing was performed only in patients with positive UAT results, with the exception of sputum specimens, introducing selection bias and precluding assessment of whether PCR provides improved diagnostic sensitivity compared with UAT in a broader, unselected patient population.

Our findings support current recommendations emphasizing lower respiratory tract specimens for molecular detection of *Legionella*. When bronchoscopy is not indicated, sputum may be a practical and potentially high-yield specimen for *Legionella* PCR, whereas NPS and urine demonstrated limited utility in this outbreak cohort. These observations require confirmation in larger studies using assays validated for sputum specimens.

## SUPPLEMENTAL MATERIAL

Supplemental material jcm.01116-26-s0001.docx Supplemental methods and Table S1.

ASM does not own the copyrights to Supplemental Material that may be linked to, or accessed through, an article. The authors have granted ASM a non-exclusive, world-wide license to publish the Supplemental Material files. Please contact the corresponding author directly for reuse.
