# Supplementary material for: Diagnostic yield of Legionella pneumophila PCR across specimen types during a community outbreak
Source: J Clin Microbiol. 2026 Oct 12;64(11):e01116-26. doi: 10.1128/jcm.01116-26 (PMC13753824; doi:10.1128/jcm.01116-26)
Supplement: Supplementary file 1 [file jcm.01116-26-s0001.docx]

**Supplemental Methods**

Nucleic acid was extracted from 200 µL of bronchoalveolar lavage (BAL), sputum, nasopharyngeal swab (NPS), and urine specimens using the NucliSENS easyMAG system (bioMérieux) according to the laboratory's standard operating procedures, with elution in 55 µL. A synthetic oligonucleotide corresponding to a bacteriophage lambda genome sequence was spiked into each specimen before extraction as an internal control at a final concentration of 5,000 copies/mL. Because of their high viscosity, sputum specimens were pre-processed before nucleic acid extraction. Briefly, 250 µL of sputum was diluted approximately fivefold in sputum liquefying solution (Copan). A 500-µL aliquot of the diluted specimen was transferred to a 2-mL tube containing a ceramic bead homogenization mix (Bertin Technologies) and homogenized for 2 minutes using a Mini Bead Mill homogenizer (VWR). Following brief centrifugation, 200 µL of the processed sputum was used for nucleic acid extraction. Urine specimens were extracted using two different methods. For the first method, 200 µL of urine was extracted directly using the NucliSENS easyMAG system. For the second method, cells were concentrated from 1 mL of urine by centrifugation at 13,000 rpm for 15 minutes. After removal of 800 µL of the supernatant, the cell pellet was resuspended in the remaining 200 µL of supernatant, and the concentrated specimen was extracted using the NucliSENS easyMAG system as described above.

Nucleic acid extracts from all specimens were tested using a laboratory-developed, TaqMan-based multiplex PCR assay that simultaneously detected *Legionella* spp., *Legionella pneumophila*, and lambda DNA as an internal control. PCR was performed according to the laboratory's standard operating procedure (SOP) using the QuantiTect® Multiplex PCR NoROX Kit (Qiagen) on a CFX Real-Time PCR Detection System (Bio-Rad). Primers and probes were used at final concentrations of 0.2 µM and 0.1 µM, respectively. Following an initial denaturation at 95°C for 10 minutes, amplification was performed for 40 cycles, each consisting of denaturation at 95°C for 30 seconds and annealing/extension at 60°C for 60 seconds. Primer and probe sequences are provided in Supplementary Table 1. A cycle threshold (Ct) value of ≤40 was considered positive. The assay was validated in-house for bronchoalveolar lavage (BAL) specimens and demonstrated a limit of detection of 10^3^ copies/mL, 100% intra-assay and inter-assay precision, and >95% sensitivity and specificity compared with the reference method [??].

**Supplemental table 1: Primers and probes used in this study**

| **Target** | **Primer/probe name** | **Sequence (5′-3′)** |
| --- | --- | --- |
| *Legionella pneumophila,* *mip* gene | LPMIPF | GATGTTARYCCGGAAGCAWTGG |
|  | LPMIPR | CGTCTTTCATTTGYTGYTCGG |
|  | LPMIPP | Quasar 670-AAGCCAATTGAGCGCCACTCATAG- BHQ2 |
| *Legionella* spp., *ssrA* gene | PanLF | ACGATGAAAACTTTGCTGGTG |
|  | PanLR/TMR | ATGACCGTTGATTCGATACCG |
|  | TMF | AACGATGAAACTTTGCTGGTG |
|  | PanLP | Cal Fluor Orange 560-TCGAACGGGATACTGGGTTTATTGGC-BHQ1 |
|  | TMP | Cal Fluor Orange 560-AAACTTTTCCCCAATCGTGCGCTG-BHQ1 |
| Lamda phage | LNFP | GCAAAAGATGAGGCCGGAGATAT |
|  | LRP | CCTTAACTTTGCCCACCT |
|  | Lambda Probe | FAM-ACCAATGCTGAGATAGCTGAAGAG-BHQ1 |
